# Unraveling the Risks: Epidemiology of Complicated Pneumococcal Bloodstream Infections

**DOI:** 10.7759/cureus.97585

**Published:** 2025-11-23

**Authors:** Nongnooch Poowanawittayakom, Rashini Jayawarden, Robin Chamberland, Christian Gill

**Affiliations:** 1 Infectious Disease, Washington University School of Medicine, St. Louis, USA; 2 Internal Medicine, Saint Louis University, St. Louis, USA; 3 Pathology, SSM Health Saint Louis University Hospital, St. Louis, USA; 4 Infectious Disease, University of Pennsylvania, Philadelphia, USA

**Keywords:** complicated bacteremia, invasive pneumococcal disease, pneumonia vaccine, risk-factors, s.pneumoniae

## Abstract

Background

Invasive pneumococcal infections, such as bacteremia, remain associated with substantial morbidity and mortality, particularly among individuals with underlying health conditions. The primary objective of this study was to evaluate the association between patient comorbidities, particularly chronic cardiac disease, and the development of complicated pneumococcal bacteremia. Secondary objectives included assessing vaccine coverage and clinical outcomes among high-risk patients.

Methods

We retrospectively reviewed medical records of patients admitted to SSM Health and St. Louis University Hospitals between 2018 and 2024 with confirmed invasive *Streptococcus pneumoniae* infection. Data collected included demographics, comorbidities, vaccination status, and clinical presentation. Complicated bacteremia was defined as bacteremia with concurrent invasive organ involvement, including endocarditis, septic arthritis/osteomyelitis, or empyema. Multivariable regression analysis was performed, with statistical significance set at P <0.05.

Results

Among 153 patients, older age, pre-existing heart disease, and congestive heart failure were significantly associated with complicated bacteremia. Common complications included endocarditis, septic emboli, empyema, and pneumonia. Laboratory markers of disease severity included hypoalbuminemia and leukocytosis. More than half of the patients with complicated bacteremia required intensive care unit admission. Vaccination rates were low among patients who developed complicated bacteremia.

Conclusions

Older age and pre-existing cardiac disease were key predictors of complicated pneumococcal bacteremia. Despite advances in vaccination and antimicrobial therapy, invasive pneumococcal disease continues to cause significant morbidity and mortality. Understanding the epidemiology and risk factors associated with complicated pneumococcal infections is critical for improving prevention, guiding early recognition, and optimizing patient outcomes.

## Introduction

*Streptococcus pneumoniae* remains a major cause of morbidity and mortality worldwide. It most commonly causes community-acquired pneumonia but can also produce a wide range of clinical syndromes [1-3]. These include asymptomatic nasopharyngeal carriage and invasive pneumococcal diseases (IPDs) such as bacteremia, meningitis, and endocarditis. The organism spreads mainly through respiratory droplets, with infection rates peaking during winter and early spring [4-6].

Both bacterial and host factors influence the persistence and severity of pneumococcal infections. The pneumococcal polysaccharide capsule and surface proteins promote nasopharyngeal colonization and help the bacterium evade host immune defenses [6]. Host-related risk factors include advanced age, immunocompromised states (such as HIV infection or hematologic malignancies), and chronic conditions like chronic lung disease, chronic kidney disease, cirrhosis, diabetes, alcoholism, and asplenia [7,8]. Socioeconomic and environmental factors, including crowding and limited access to healthcare, further increase susceptibility. Older adults face a higher risk not only because of comorbidities but also due to waning immunity and reduced vaccine responsiveness [9].

Although uncommon, pneumococcal endocarditis causes severe illness and high mortality. Because its presentation often lacks the classic features of infective endocarditis, clinicians may delay diagnosis and treatment. These delays increase the risk of valve destruction, abscess formation, systemic embolization, and death, with case fatality rates ranging from 28% to 60%. We classified patients as having complicated or uncomplicated pneumococcal bacteremia. We defined complicated bacteremia as bacteremia with a concurrent invasive source, including endocarditis, septic arthritis/osteomyelitis, or empyema.

Pneumococcal vaccination remains the cornerstone of prevention and is recommended for all age groups, particularly for children, older adults, and individuals with underlying conditions [7]. Clinicians typically treat pneumococcal infections with intravenous or oral beta-lactam antibiotics, such as ceftriaxone or penicillin. When resistance or allergies occur, alternatives such as levofloxacin, vancomycin, or linezolid may be appropriate [7].

## Materials and methods

Study design and population

This study was approved by the Institutional Review Board in 2024 under exempt status, as it posed no more than minimal risk to participants. We conducted a retrospective cohort study of 296 patients with *Streptococcus pneumoniae* bacteremia, admitted to SSM Health and Saint Louis University Hospital between January 1, 2018, and February 28, 2024. Of these, 153 (51.7%) developed complicated bacteremia. Eligible patients had documented *S. pneumoniae* bacteremia confirmed by positive blood cultures during the study period.

Data collection and abstraction

A single trained reviewer abstracted data using a standardized and pre-tested electronic data collection form, designed to minimize variability and bias. The form included predefined fields and operational definitions for all study variables. To ensure reliability and reproducibility, an independent reviewer cross-checked a random 10% subset of records for internal consistency and accuracy.

Extracted variables included demographics (age, sex), immunocompromised status, vaccination history, comorbidities, antibiotic regimens, and treatment duration. Major comorbidities, such as chronic heart disease, congestive heart failure, chronic lung disease, diabetes, liver disease, and renal disease, were defined using ICD-10 (10th revision of the International Classification of Diseases) codes recorded in the electronic medical record. Clinical outcomes assessed included 30-day mortality and in-hospital mortality. Missing data were handled by listwise deletion, and the extent of missingness was reviewed to confirm that it occurred at random.

Microbiology data and β-lactam resistance

Microbiologic data were obtained from institutional clinical microbiology laboratory databases. *Streptococcus pneumoniae* isolates were identified using standard microbiological methods, including automated systems or matrix-assisted laser desorption/ionization time-of-flight (MALDI-TOF) mass spectrometry. Antimicrobial susceptibility testing (AST) was performed using broth microdilution or automated platforms in accordance with Clinical and Laboratory Standards Institute (CLSI) guidelines.

β-lactam resistance was defined according to CLSI systemic and meningitis breakpoints. Isolates were considered β-lactam-resistant if they demonstrated intermediate or resistant non-susceptibility to at least one of the following agents: penicillin, ceftriaxone, or cefotaxime. Results were validated against laboratory quality control standards and verified by senior microbiology personnel before inclusion in the dataset.

Definition of complicated bacteremia

Patients were classified as having complicated or uncomplicated pneumococcal bacteremia. Complicated bacteremia was defined as bacteremia with concurrent invasive organ involvement, including endocarditis, septic arthritis/osteomyelitis, or empyema.

Vaccination data

Vaccination records were abstracted from the electronic medical record, assessing receipt of PCV13, PPSV23, and PCV20. Vaccine eligibility was determined according to Advisory Committee on Immunization Practices (ACIP) adult pneumococcal vaccination guidelines, defined as age ≥50 years or age 19-49 years with one or more of the following risk factors: alcohol abuse; chronic heart, liver, or lung disease; asplenia; malignancy; diabetes mellitus; solid organ transplant; immunosuppression; or sickle cell disease. Full vaccination was defined as receipt of one dose of PCV13 followed by two doses of PPSV23, or a single dose of PCV20.

Statistical analysis

Bivariate analyses identified risk factors for complicated IPD. Categorical variables were summarized as numbers (percentages) and compared using the χ² test or Fisher’s exact test, as appropriate. Continuous variables were reported as median (interquartile range (IQR)) and compared using the Mann-Whitney U test. All statistical analyses were conducted using SPSS version 25 (IBM Corp., Armonk, NY). A two-sided P-value <0.05 was considered statistically significant.

## Results

Patient characteristics

Table 1 summarizes the baseline characteristics of 296 patients with *S. pneumoniae* bacteremia, 153 (51.7%) of whom developed complicated bacteremia. Patients with complicated bacteremia were older than those without (median 64 years (IQR 19) vs. 60 years (IQR 20), P = 0.002). Other baseline characteristics, including sex, body mass index, race, housing status, and residence in long-term care facilities, were similar between groups. Among comorbidities, pre-existing cardiac disease was more common in patients with complicated bacteremia (38.6% vs. 24.0%, P = 0.007), as was congestive heart failure (28.1% vs. 15.0%, P = 0.022). Other underlying medical conditions, including diabetes mellitus, chronic obstructive pulmonary disease, asthma, cancer, end-stage liver or renal disease, autoimmune disease, and HIV, were similar between groups. Substance use and smoking history did not differ significantly.

**Table 1:** Baseline Demographics and Comorbidities of Patients with Complicated vs. Uncomplicated Bacteremia Continuous variables are presented as median (interquartile range, IQR); categorical variables are presented as numbers (percentages). P-values were calculated using the Mann–Whitney U test for continuous variables and chi-square or Fisher’s exact test for categorical variables as appropriate. CAD, coronary artery disease; CHF, congestive heart failure; COPD, chronic obstructive pulmonary disease.

Characteristic	Complicated Bacteremia (N = 153)	Uncomplicated Bacteremia (N = 143)	P-Value
Continuous Variables			
Age, years	64 (55-74)	60 (47-67)	0.002
BMI, kg/m²	25 (22-31)	26 (22-33)	0.166
Categorical Variables			
Female sex	82 (53%)	67 (47%)	0.297
Race			0.196
White/Caucasian	101 (66%)	84 (59%)	
African American	47 (31%)	55 (38%)	
Asian	1 (0.7%)	1 (0.7%)	
Hispanic/Latino	0 (0%)	2 (1%)	
Multiracial	3 (2%)	0 (0%)	
Unhoused	5 (3%)	7 (4.9%)	0.463
Long-term care facility residence	5 (3%)	8 (5.6%)	0.316
Cardiac disease (CAD, valvular disease, CHF)	59 (38.6%)	34 (23.8%)	0.007
Congestive heart failure	43 (28%)	21 (14.7%)	0.022
Diabetes mellitus	45 (29.4%)	40 (28%)	0.844
COPD	50 (33%)	34 (23.8%)	0.097
Asthma	30 (19.6%)	20 (14%)	0.207
Cancer (any)	41 (26.8%)	32 (22.4%)	0.146
End-stage liver disease	3 (2%)	1 (0.7%)	0.351
End-stage renal disease	10 (7%)	9 (6%)	0.945
Autoimmune disease	10 (7%)	9 (6%)	0.946
HIV	7 (5%)	9 (6%)	0.504

Clinical manifestations and outcomes

Complicated bacteremia was associated with multiple invasive manifestations, including empyema (144 patients, 94.1%), endocarditis (15 patients, 9.8%), septic arthritis (six patients, 3.9%), and septic emboli (six patients, 3.9%). Pneumonia occurred more frequently in complicated cases than in uncomplicated bacteremia (90.8% vs. 75.0%, P < 0.001). Laboratory findings demonstrated that patients with complicated bacteremia had lower median albumin levels (3.0 g/dL (IQR 0.9) vs. 3.4 g/dL, P = 0.005) and higher median white blood cell counts (15.1 × 10⁹/L (IQR 13.8) vs. 11.5 × 10⁹/L, P = 0.005). ICU admission was required in 54.9% of complicated cases, reflecting greater illness severity (P = 0.002).

Vaccination indications and status

Most patients met the indications for pneumococcal vaccination according to ACIP guidelines (93% of complicated vs. 91% of uncomplicated cases). Despite this, full vaccination was low, with only 12% of complicated and 13% of uncomplicated patients fully vaccinated, while approximately half of each group had received at least one pneumococcal vaccine (Table 2).

**Table 2:** Vaccination Status, Antimicrobial Therapy, and Microbiology Percentages are calculated relative to the total cohort in each group (N=153 for complicated, N=143 for uncomplicated). Oral β-lactam percentages are calculated among patients who received oral therapy. Resistance breakpoints are reported according to CLSI guidelines for meningitis and systemic isolates. “—” indicates not applicable or P-value not calculated. ACIP, Advisory Committee on Immunization Practices; CLSI, Clinical and Laboratory Standards Institute.

Variable	Complicated Bacteremia (N = 153), n (%)	Uncomplicated Bacteremia (N = 143), n (%)	P-Value
Pneumococcal vaccine indication (per ACIP)	142 (93)	133 (93)	0.521
Fully vaccinated	18 (12)	19 (13)	0.812
At least one vaccine	76 (50)	73 (51)	0.998
IV Ceftriaxone	118 (77)	129 (90)	0.023
Oral switch	72 (47)	74 (52)	0.645
Oral β-lactam among oral therapy	55 (76)	50 (68)	0.187
β-lactam resistance	28 (18)	25 (18)	0.839
Penicillin-meningitis breakpoint resistance	28 (18)	23 (16)	—
Ceftriaxone-meningitis breakpoint resistance	10 (6.5)	7 (4.9)	—
Cefotaxime-meningitis breakpoint resistance	7 (4.6)	5 (3.5)	—
Systemic breakpoint resistance	2 (1.3)	0 (0)	—

Mortality

Thirty-day mortality was higher in complicated versus uncomplicated bacteremia (13% vs. 8%, P = 0.103), whereas in-hospital mortality was similar (20% vs. 16%, P = 0.299). Among patients with endocarditis (10% of bloodstream infections (BSIs)), 30-day mortality was 27%. Receipt of oral therapy after hospital discharge did not significantly affect 30-day mortality in either group (complicated: 13% vs. 21%, P = 0.24; uncomplicated: 7% vs. 10%, P = 0.57). Oral β-lactam therapy was not associated with increased 30-day mortality compared with other oral agents or IV therapy (10% vs. 10%, P = 0.927).

## Discussion

Invasive pneumococcal infections, particularly bacteremia, remain a significant clinical challenge, affecting 20-25% of patients and carrying a mortality rate of 20-30% [4,5]. Despite advances in vaccination and antimicrobial therapy, older adults and patients with pre-existing cardiac conditions experience disproportionately severe outcomes. Our study identifies advanced age, underlying heart disease, and congestive heart failure as significant predictors of complicated bacteremia, highlighting the need for tailored preventive and management strategies for high-risk populations. Clinically, complicated cases frequently present with endocarditis, septic emboli, empyema, and pneumonia, accompanied by laboratory markers such as hypoalbuminemia and leukocytosis. The need for ICU-level care and prolonged treatment underscores the severe clinical trajectory in these patients.

Mechanistically, pre-existing cardiac disease may predispose to complicated bacteremia through multiple pathways. Chronic heart disease and heart failure reduce systemic perfusion and tissue oxygenation, impairing innate immune defenses and facilitating bacterial proliferation. Hemodynamic stress, endothelial dysfunction, and systemic inflammation may also increase vascular permeability, promoting bacterial translocation into the bloodstream. These factors collectively create an environment in which *S. pneumoniae* can more readily invade the circulation, leading to life-threatening complications.

Vaccination remains a cornerstone for preventing IPD. PPSV23 and PCV13 reduce the incidence of pneumococcal pneumonia and may curb antimicrobial resistance [1,3,10-15]. In our cohort, vaccination was not associated with a reduced risk of complicated bacteremia, likely due to low overall uptake and limited coverage of PCV20 during the study period. These findings align with prior reports of suboptimal adult pneumococcal vaccination in the US [16-18]. Prevention strategies, especially among the elderly, should focus on maximizing vaccination according to ACIP recommendations, including PCV20 or PCV15, followed by PPSV23. Early identification and management of comorbidities, along with lifestyle interventions such as smoking cessation, nutrition optimization, and physical activity, can further reduce susceptibility. Institutional measures, including nurse-led vaccination programs, pharmacist-driven initiatives, and electronic health record reminders, are critical for improving adherence among high-risk patients [19,20].

Regarding therapy, IV β-lactams were standard, with nearly 50% of patients transitioned to oral antimicrobials after initial IV therapy. Prior studies support the safety and efficacy of partial oral regimens for uncomplicated streptococcal bacteremia [10-12]. Our study extends this observation to complicated bacteremia, including empyema and septic arthritis, showing oral β-lactams were used safely without increasing 30-day mortality. These findings suggest that oral β-lactams may be a viable alternative to fluoroquinolones, given their favorable pharmacokinetics and low minimum inhibitory concentrations (MICs) against *S. pneumoniae*, although larger prospective studies are warranted [13,14].

Approximately 50% of BSIs had an identifiable focus associated with complicated disease, including endocarditis in 5% of cases. Despite >90% of patients meeting ACIP vaccination criteria, only 12-13% were fully vaccinated, emphasizing persistent gaps in adult immunization.

Limitations

This study has several limitations. First, its retrospective design limits causal inference. Second, reliance on medical records introduces potential information bias, and missing or incomplete vaccination data may affect accuracy. Third, single-reviewer data abstraction, despite internal validation, may reduce reliability. Fourth, exploration of confounding factors beyond bivariate analysis was limited. Fifth, rare risk factors may have been missed, and the small sample size limited statistical power. Sixth, as a single-center study, generalizability is restricted. The authors acknowledge these limitations transparently, reinforcing the need for cautious interpretation.

Despite these constraints, the study provides actionable insights: clinicians should maintain heightened vigilance for older adults and patients with cardiac comorbidities, monitor laboratory indicators such as albumin and WBC counts as early signs of complicated disease, and prioritize vaccination and comorbidity management to prevent invasive pneumococcal infections.

## Conclusions

This study identifies older age and pre-existing cardiac comorbidities as key risk factors for the development of complicated *S. pneumoniae* bacteremia. These findings underscore the need for targeted preventive strategies in high-risk populations to mitigate progression to severe invasive disease. While the study was underpowered to assess vaccine effectiveness due to low vaccination uptake, our results highlight the urgent need to improve vaccination coverage among vulnerable patients.

Additionally, observational data support the feasibility and potential safety of oral β-lactam therapy in both uncomplicated and complicated cases, though prospective studies are needed to confirm these findings. Collectively, these data provide clinically actionable insights, inform risk stratification, and guide future research aimed at reducing morbidity and mortality associated with pneumococcal bacteremia.

## References

[1] Gierke R, Wodi P, Kobayashi M (2024). Chapter 17: Pneumococcal disease. Epidemiology and Prevention of Vaccine-Preventable Diseases.

[2] Ali S, Kim P, Dhar S (2023). Pneumococcal endocarditis, rare but not forgotten. Ann Intern Med Clin Cases.

[3] Dion CF, Ashurst JV (2023). Streptococcus pneumoniae. StatPearls [Internet].

[4] Musher D (2025). Invasive pneumococcal (Streptococcus pneumoniae) infections and bacteremia in adults. UpToDate.

[5] Robinson KA, Baughman W, Rothrock G (2001). Epidemiology of invasive Streptococcus pneumoniae infections in the United States, 1995-1998: Opportunities for prevention in the conjugate vaccine era. JAMA.

[6] Tuomanen E, Musher D (2025). Streptococcus pneumoniae: Microbiology and pathogenesis of infection. UpToDate.

[7] Smith DA, Nehring SM (2023). Bacteremia. StatPearls [Internet].

[8] Ditzel K, Giardina F, ten Oever J, Cremers AJH (2025). Risk conditions for invasive pneumococcal disease in adults: A systematic review and meta-analysis. EClinicalMedicine.

[9] Rueda AM, Serpa JA, Matloobi M, Mushtaq M, Musher DM (2010). The spectrum of invasive pneumococcal disease at an adult tertiary care hospital in the early 21st century. Medicine (Baltimore).

[10] Lindberg J, Prag J, Schønheyder HC (1998). Pneumococcal endocarditis is not just a disease of the past: An analysis of 16 cases diagnosed in Denmark 1986-1997. Scand J Infect Dis.

[11] Lefort A, Mainardi JL, Selton-Suty C, Casassus P, Guillevin L, Lortholary O (2000). Streptococcus pneumoniae endocarditis in adults. A multicenter study in France in the era of penicillin resistance (1991-1998). The Pneumococcal Endocarditis Study Group. Medicine (Baltimore).

[12] de Egea V, Muñoz P, Valerio M (2015). Characteristics and outcome of Streptococcus pneumoniae endocarditis in the XXI century: A systematic review of 111 cases (2000-2013). Medicine (Baltimore).

[13] Sims RV, Steinmann WC, McConville JH, King LR, Zwick WC, Schwartz JS (1988). The clinical effectiveness of pneumococcal vaccine in the elderly. Ann Intern Med.

[14] Shapiro ED, Berg AT, Austrian R (1991). The protective efficacy of polyvalent pneumococcal polysaccharide vaccine. N Engl J Med.

[15] Torres A, Blasi F, Dartois N, Akova M (2015). Which individuals are at increased risk of pneumococcal disease and why? Impact of COPD, asthma, smoking, diabetes, and/or chronic heart disease on community-acquired pneumonia and invasive pneumococcal disease. Thorax.

[16] Chinchilla AH, Melton TC, Westrick SC, Hastings TJ, Vieira L, Marley GT, Carpenter DM (2025). A cross-sectional survey to identify current pneumococcal vaccination practices and barriers in rural community pharmacies. Vaccines (Basel).

[17] Fukuda H, Onizuka H, Nishimura N, Kiyohara K (2022). Risk factors for pneumococcal disease in persons with chronic medical conditions: Results from the LIFE Study. Int J Infect Dis.

[18] Chen H, Matsumoto H, Horita N, Hara Y, Kobayashi N, Kaneko T (2021). Prognostic factors for mortality in invasive pneumococcal disease in adult: A system review and meta-analysis. Sci Rep.

[19] Moberley S, Holden J, Tatham DP, Andrews RM (2013). Vaccines for preventing pneumococcal infection in adults. Cochrane Database Syst Rev.

[20] Bonten MJ, Huijts SM, Bolkenbaas M (2015). Polysaccharide conjugate vaccine against pneumococcal pneumonia in adults. N Engl J Med.

